# Occurrence and Health Effects of Hexabromocyclododecane: An Updated Review

**DOI:** 10.3390/toxics11050409

**Published:** 2023-04-26

**Authors:** Maria Lopes Marques, Elisa Cairrao

**Affiliations:** 1 CICS-UBI, Health Sciences Research Centre, University of Beira Interior, 6200-506 Covilhã, Portugal; 2 FCS-UBI, Faculty of Health Sciences, University of Beira Interior, 6200-506 Covilhã, Portugal

**Keywords:** persistent organic pollutant, bromine flame retardant, HBCD, endocrine disrupting chemicals (EDC), environmental toxicity, human toxicity

## Abstract

Hexabromocyclododecane (HBCD) is a non-aromatic compound belonging to the bromine flame retardant family and is a known persistent organic pollutant (POP). This compound accumulates easily in the environment and has a high half-life in water. With a variety of uses, the HBCD is found in house dust, electronics, insulation, and construction. There are several isomers and the most studied are α-, β-, and γ-HBCD. Initially used as a substitute for other flame retardants, the polybrominated diphenyl ethers (PBDEs), the discovery of its role as a POP made HBCD use and manufacturing restricted in Europe and other countries. The adverse effects on the environment and human health have been piling, either as a result from its accumulation or considering its power as an endocrine disruptor (ED). Furthermore, it has also been proven that it has detrimental effects on the neuronal system, endocrine system, cardiovascular system, liver, and the reproductive system. HBCD has also been linked to cytokine production, DNA damage, increased cell apoptosis, increased oxidative stress, and reactive oxygen species (ROS) production. Therefore, this review aims to compile the most recent studies regarding the negative effects of this compound on the environment and human health, describing the possible mechanisms by which this compound acts and its possible toxic effects.

## 1. Introduction

**Figure 1:**
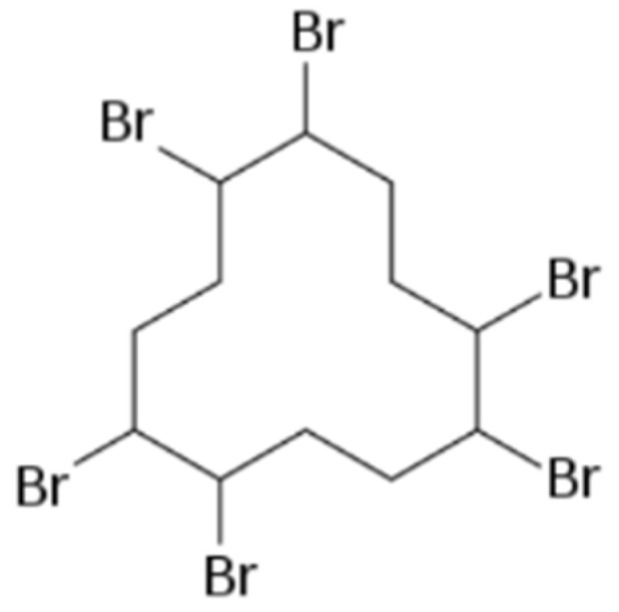
Structure of HBCD, created with BioRender.comb (accessed on 26 March 2023).

Hexabromocyclododecane (HBCD) (Figure 1) is a part of bromine flame retardants (BFR) and a non-aromatic compound portrayed as an additive retardant [1,2]. Being a compound that does not bind chemically, it is primarily found as an addition to polymers, such as polystyrene foams, building materials, plastics, or some organic materials [3,4], and secondarily in electronics or car interiors, such as car cushions [5]. It can free itself from its products, accumulating in the environment.

HBCD is highly lipophilic, with a low solubility in water, and aliphatic [6,7] with a higher half-life in water than in the air [8]. HBCD has 16 stereoisomers [6] and can be a result from BFRs production and consumer products wrongful disposal with concomitant leaching. This high number of stereoisomers creates diverse paths for exposure [9].

Through its abundance in stereoisomers, HBCD can have several different isomer concentrations, depending on its final destination and its respective production process, since it can undergo rearrangement at temperatures above 160 °C [9,10].

From 1960 to 2009, the use of HBCD was mainly linked to the construction industry. In 2009, its production and use were restricted. By 2013, HBCD became part of the Stockholm Convention’s Annex A as a persistent organic pollutant [4]. Consequently, concentration limits were imposed in 2019 by the European Union for products placed in the market and the ones entering waste streams. Moreover, the limit for HBCD disposal was also established as 1000 mg/kg [11]. Until the end of 2019, HBCD’s production and use, in circumstances, such as domestic insulation, was still permitted. However, on the 26 November 2019, its use was prohibited in the European Union [11]. From this point onwards, HBCD was considered a persistent organic pollutant (POP) with high bioaccumulation, long-distance transport and associated high toxicity [4,12]. POPs are found in any environment, in the air, soil, and in the aquatic environment, but their risks are not yet fully described. Nevertheless, some studies prove the negative effects of HBCD on plants, animals and humans [9].

Continuous exposure to HBCD affects various systems, including increased liver weight and liver enzyme, and additional side-effects on the thyroid hormone. Furthermore, this exposure can affect the reproductive system and induce alterations in spontaneous behavior and memory defects. Studies in vitro show that HBCD has anti-androgen activity and can be an aromatase inhibitor, interacting with steroid hormone receptors. These effects show that HBCD can be an endocrine disruptor, causing neuronal changes in animals and affecting the hormonal system, along with several other systems [13,14,15].

This review summarizes the most important and relevant studies about the mode of action and effects of HBCD on the environment and human health. In addition, it intends to describe the effects on the various systems and metabolic pathways of plants, animals, and humans. This work highlights the importance of continued studies involving HBCD, focusing on its dangers, and stereoisomers. The main topics covered by this review are exposure routes; toxicity studies; and the effects on human health, animals, and plants. For this purpose, research was carried out on chemical properties and routes of exposure of the HBCD, and all the in vitro and in vivo studies performed in all the organisms (plants, animals, and humans), as to more rigorously assess safety and the benefits or hazards related with the usage of HBCD.

## 2. Approach to the Review

This review summarized experimental studies that evaluated the effects of HBCD in plants (Topic 4), animal (Topic 5), and human models (Topic 6) after 2014. Furthermore, the HBCD’s effects in several body systems were also explored, mainly focusing on the cardiovascular, reproductive, endocrine, neuronal, and liver.

A literature review was performed based on articles available in Scopus, Web of Science, PubMed, and ScienceDirect (Elsevier) databases. The research strategy was carried out using Boolean operators “AND”, “OR”, and “NOT” and a combination of terms relating to HBCD, such as “tHBCD”, “Hexabromocyclododecane”, “α-HBCD”, “β-HBCD”, and “γ-HBCD”. This article was conducted following the protocol used by Abrantes-Soares et al. [16]. In addition to these terms, search relevant citations of the articles retrieved were also included.

## 3. Occurrence and Exposure to HBCD

HBCD was first found in the Swedish rivers in 1997 [17]. After its discovery, this compound was found in numerous different ecosystems and natural resources [18]. Due to its low solubility and vapor pressure, HBCD has a high bio-accumulative potential and can enter long-range transport [19]. Moreover, De Wit et al., 2010, found HBCD in the Arctic, which corroborated the suspicion of long-range airborne transport of this compound [20]. In the same year, HBCD levels in Europe came below the detection limit and a maximum value of 0.00266 g/Kg dry weight (dw) was found in the river Ebro, in northern Spain [21,22]. Despite this, these concentrations were much higher than those seen in North America and Southwest Asia, which was justified by the high production of this compound in Europe before restrictions were imposed by the European Union. Furthermore, Zang et al., 2013, found that HBCD values increased substantially close to emission points, such as extruded polystyrene (XPS-) and expanded polystyrene (EPS-) production plants, having a significant presence in the Northern Hemisphere [23]. Some studies point to the concentration stabilization of HBCD in the environment in recent years [24,25]. On the other hand, other studies show that HBCD concentrations detected have increased in humans [26,27].

Concerning the HBCD structure, Wu et al., 2012, and Heeb et al., 2007, describe that the technical HBCD (tHBCD), synthesized from the addition of bromine at 1,5,9-cyclododecatriene, consists of a mixture of three diastereomers: α-, β-, and γ-HBCD. In addition to these, other diastereomers, such as δ-HBCD, were found in abiotic [28] and biotic matrices [29,30,31], but most research has been conducted in α-, β-, and γ-HBCD. Moreover, Wu et al., 2012, and Heeb et al., 2007, also concluded that the amount of each of these diastereomers varied within a range of available products, but with trends for 1–12% of α-, 10–13% of β-, and 75–89% of γ-HBCD [32,33].

With the composition of abiotic matrices similar to the commercial mixture, and γ-HBCD as the most abundant diastereomer, Zhang et al., 2013, suggested the use of a ratio of γ- and α-HBCD in abiotic samples for the management of HBCD environmental contamination, since γ-HBCD tends to decline over time [23]. However, Li et al., 2012, and Meng et al., 2012, found that the diastereomer α-HBCD is rather the most abundant in abiotic systems [28,34]. Law et al., 2014, detected time trends of environmental concentrations of HBCD with increases and decreases in different matrices [35]. There are also several works describing that γ-HBCD has a faster metabolism than α-HBCD, which justifies the increase in α-HBCD over time and the consequent decrease in γ-HBCD [9,18].

In agreement, in 2012, Du et al. exposed zebrafish to three diastereomers of HBCD (α-, β-, and γ-), to determine their effects on the organism of these animals [36]. The authors concluded that HBCD bioaccumulation was dependent on the concentration to which the animal was exposed, and that the α-HBCD diastereomer had the longest half-life, greater accumulation efficiency and greater number of biomagnification factors, reinforcing the idea that α-HBCD would be the most prevalent diastereomer in biota samples, similar to some studies already published in 2011 [5,37]. Beyond that, Badea et al., 2016, reinforces that most studies conducted in fish, rats, birds, and humans report an enantioselective absorption, metabolism, and excretion of α-HBCD [38].

Domestic dust, and indoor and outdoor airborne concentrations of HBCD have gathered special attention since they are the major human health influencers. Several scientists have led a series of studies using air and dust samples and hand wipes to determine the levels of HBCD contamination to which a human may be exposed without endangering human health. Harrad et al., 2010, reported high levels of HBCD exceeding 0.01 g/Kg in daycare centers and primary schools in the UK [39]. Abdallah et al., 2008, and De Wit et al., 2012, found higher HBCD concentrations in public indoor environments in the UK, with a maximum of 0.0032 g/Kg and a maximum of 0.14 g/Kg in houses [40,41]. Tests using hand wipes performed in the USA demonstrated low levels of HBCD with values between 5 × 10^−14^ Kg and 1.08 × 10^−11^ Kg [42]. HBCD presence was also found in dust samples in urban and rural areas in Sweden (between 3.12 × 10^−18^ mol/L and 9.51 × 10^−16^ mol/L) [9]. Additionally, HBCD concentration was higher than that of PDBEs in sediments and suspended particulate material (SPM) downstream of urban centers and industrial areas. The highest concentrations of SPMs (0.017 g/Kg dry weight) were downstream of HBCD production sites, such as rivers in UK and Belgium [43], and downstream of industrial infrastructures insulated with HBCD for Sweden [17] and Spain [44]. Soils surrounding HBCD processing factories also contained high levels of HBCD between 1.11 × 10^−4^ g/Kg and 0.032 g/Kg dry weight [9].

The occurrence of HBCD in sewage sludge [43,45,46,47] becomes a consequence of diffuse leaching and abrasion of products containing flame retardants in wastewater and streams [47]. The use of these sludges in agriculture can redistribute HBCD to soil, aquatic, terrestrial, and food chain environments, as seen with PDBEs [48,49]. In the United States, after the ban on the production of PDBEs in 2004, HBCD levels in fish increased. A noteworthy example is the carp of Hyco River: by 2002, the average HBCD concentration was of 1.3 × 10^−5^ g/Kg lw and by 2007, this value increased to an average concentration of 0.00501 g/Kg lw [50]. This suggests that the termination of the use of PDBEs caused an increase in the use of HBCD as an alternative, which, in turn, led to an increase in its appearance in the surrounding environments.

The presence of HBCD in vegetables has also been observed and can be correlated with the use of sewage sludge as fertilizer [51]. Wu et al., 2012, proved the accumulation of HBCD-specific diastereomers in corn, which, as a consequence, caused oxidative stress and DNA damage [32]. Li et al., 2011, showed that an increase in HBCD concentration in plants and in the human diet was limited to absorption in the soil matrix [52]. The same author described higher concentrations of HBCD in root tissue than in shoot tissue for radish cabbage. The dominant diastereomer in root tissue was γ-HBCD while α-HBCD was dominant in shoot tissue. These results may be explained by the water solubility of each diastereomer, being higher for α-HBCD compared to γ-HBCD [53]. Zhang et al., 2013, demonstrated that plants that are not created in the laboratory but collected from the fields had a higher concentration of HBCD in the leaves rather than in the roots, most likely due to air absorption, explaining why the diastereomer γ-HBCD was the one with the highest concentration in this study [54]. Although studies in plants are scarce, for animals, the number of studies is already considerable, particularly in birds and aquatic organisms [35].

Fish typically exhibit high HBCD levels, given their position in the food chain and their high exposure in aquatic environments [9]. Higher concentration of HBCD were found in fish downstream of factories in the UK [55] than fish upstream, with the same effect observed in Spain and Sweden [9]. Meng et al., 2012, discovered much higher HBCD concentrations in freshwater farmed fish than in marine fish, pointing to the influence of human activities in the level of HBCD in aquaculture [34]. HBCD levels were found in two species of fish from nine coastal cities in China. The findings reported levels of HBCD contamination in all coastal cities where the analysis was performed. Despite the levels of contamination, the values were low compared to the values in Europe, justified by the excessive use of HBCD in Europe [56]. Nakagawa et al., 2010, analyzed several samples of wild sea life and farmed seafood in Japan and concluded that the low levels of HBCD were similar to those found in China [57].

Janak et al., 2005, first reported the presence of HBCD in marine mammals of the Western Scheldt estuary [29]. Three years later, the same group detected a diastereomeric composition of HBCD in eggs of peregrine falcons, white-tailed sea eagles, guillemots, and common terns. A high fraction of (+)-α-HBCD was found in white-tailed sea eagles, while peregrine falcons and common terns had preferentially (−)-α-HBCD [30]. Guerra et al., 2012, described a pattern of HBCD isomers in some, but not all, birds with dominance in α-HBCD. Birds of prey were the species with the highest HBCD concentration, being most notable in the falcon egg peregrine of Montreal, Canada, with α-HBCD being the most dominant [58]. Leslie et al., 2011, found a dominance of γ-HBCD in 1995 in the sparrowhawk muscle in the UK [59].

Regarding human exposure to HBCD, dermal and oral are the main routes of exposure, but inhalation of vapor or particles was also an important source of HBCD [5]. Industrial plant workers producing EPS with HBCD were suspected to be one of the groups with major concentrations of HBCD, given their direct contact with skin and inhalation of small particles. High levels of HBCD in the blood were found, ranging between 6 × 10^−6^ g/Kg and 8.56 × 10^−4^ g/Kg lipids, respectively [60]. HBCD values in the human diet vary globally and regionally with no defined pattern [61,62]. Foods, such as fish and meat, are probably the greatest source of exposure in the human diet and the concentrations found are dependent on consumption trends [61,63] and the highest concentrations are found in fish [27]. At the human level, this compound was mainly present in breast milk, urine and blood. In breast milk samples, both high concentrations and low HBCD concentrations were found, the highest being 2.7 × 10^−5^ g/Kg [64], where the dominant diastereomer was γ-HBCD. In human blood studies, some of the concentrations found of HBCD were 1.7 × 10^−6^ g/Kg [62], 1.1 × 10^−6^ g/Kg [65] and 4.6 × 10^−7^ g/Kg [66] with α-HBCD as the dominant diastereomer.

Since exposure to HBCD is high and comes from various pathways, it has already been found in adipose tissue [67] and human blood [60,62,65]. Several studies conducted in rats have shown that HBCD has toxic potential for the thyroid [5], particularly in humans [68]. The HBCD concentrations described for the biota are usually defined in an animal as a whole or relating to tissues, such as muscle or adipose tissue. However, these determinations may have less relevance for determining effects in several organs, such as the liver, thyroid, or even nerve tissue [5].

In summary, HBCD is still very prevalent in the environment, although its production and application are banned, which promotes significant human exposure. In addition, this compound presents a high bioaccumulation, promoting high biomagnification, along the entire trophic chain. Therefore, it is very important to understand the effects of this compound on plants, animals, and humans, and all the systems and mechanistic pathways that can affect the ecosystem.

## 4. Studies in Plants

Regarding plant studies, as mentioned earlier, this literature review only looks at articles after 2014. Therefore, this review will address the four articles found and summarized in Table 1.

In 2016, Wu et al. analyzed the effect of HBCD on maize and discovered that the concentrations of this flame retardant were different for various parts of the plant. They then discovered that after the first HBCD uptake, the concentration of HBCD was higher in the roots than in the shoots. The highest concentrations, both in roots and shoots, of diastereoisomers were, in order, γ-HBCD, followed by β-HBCD and α-HBCD, which was the least concentrated. This shows that γ-HBCD is the most abundant diastereoisomer in plants samples. In addition, it has also been shown that exposure to this toxicant decreases the germination rate, root biomass, root elongation, biomass of shoots, and even the elongation of shoots. The HBCD also caused an increase in the level of hydroxy radical (OH·) in the roots and maize shoots, being higher in shoots. This can lead to oxidative stress and ROS formation and these levels are derived from HBCD exposure. They also found high values of γ-H2AX, suggesting that the damage to maize DNA would be very serious after exposure to HBCD [69].

In the same year, Huang et al. studied the accumulation and biotransformation of HBCD in the roots of maize. All three HBCD enantiomers were found in maize roots and their accumulation was diastereomer- and enantiomer-specific, with γ-HBCD being the most concentrated. They found that isomerization occurred for all stereoisomers except (+) α-HBCD. Several metabolites of HBCD were found in the roots of maize, which did not appear in nutrient solutions or in the shoots and further demonstrates that its metabolization occurred. Debromination and hydroxylation were the two ways of metabolism for HBCD. All pairs of enantiomers could bind to various active cavities of maize enzymes CYP71C3v2 and glutathione s-transferase (GST) GST31. These findings suggest that the biotransformation of HBCD enantiomers may derive from their binding to different binding sites with maize enzymes, as (−) α-HBCD formed one halogen bond with residues of CYP71C3v2, such as Arg473 and Cys476, and (+) α-HBCD formed one halogen bond with Asn326 residue. With GST31 residues only (−)α-HBCD formed halogen bonds with the residue Ser14 [70].

In 2018, Zhang et al. decided to use Pak Choi leaves to demonstrate the effects of HBCD. The analysis was performed on changes in the concentration of metabolites and showed that, in the presence of HBCD, several metabolic pathways were affected, such as the lipid, nucleotide, amino acid, and carbohydrates. The results also showed, for the first time, that HBCD can interfere with the biosynthesis of other secondary metabolites, with significant exchanges in eight metabolites: luteolin, rutin, caffeic acid, sinapic acid, conifer aldehyde, chlorogenic acid, and coniferyl. These secondary metabolites can direct and indirectly react. In addition, 13 metabolites with the power to be potential biomarkers, used to differentiate the presence of HBCD toxicity in plant leaves, were discovered [71]. Even with these results, the authors reinforce the idea that it is difficult to elucidate the mechanism of HBCD toxicity only with information on metabolites and more studies involving metabolomics and lipidomics are needed to fully understand these toxicity mechanisms.

In 2019, Huang et al. using maize microsomes analyzed if microsomal cytochrome P450 (CYPs) were involved in the degradation of HBCD through an in vitro and silico study. The findings described that after 120 min of incubation, HBCD levels were lower compared to the initial levels. Since they did not detect γ-HBCD-derive from α-HBCD, the authors suggested that there was no bio-isomerization of HBCD catalyzed by maize CYPs. The appearance of several HBCD metabolites confirmed that maize CYPs were involved in HBCD metabolism. They also showed that dependent on the diastereomers of HBCD, there were selective modifications in the protein content of CYP, and the activity of CYP. Thus, (−)/(+) α-HBCD significantly decreased the maize CYP protein level and enzyme activity, and (−)/(+) γ-HBCD were inducers of maize CYPs. These results suggest that HBCD has a disruptor power in the CYP system of maize, both at the protein content level and at the transcription level. Several CYP isoforms were altered after exposure to HBCD showing different catalytic activities which, alongside the presence of hydroxylated metabolites, suggests that these isoforms may be involved in HBCD metabolism. Despite these findings, the authors described that the mechanisms by which this biotransformation by CYPs occurs are not yet clear due to the absence of pure plant CYP isoforms, highlighting the need to verify how HBCD enantiomers enter the CYPs and how metabolic products are released [72].

In conclusion, effects of HBCD in plants can be attributed to alterations in the activity of CYPs, suggesting that the CYPs can be involved in the biodegradation of HBCD, modifying the rate of germination of the plant, leading to oxidative stress, and affecting various metabolic pathways.

## 5. Studies in Animal Models

Animal studies in this review, considering both in vitro and in vivo studies, are summarized in Table 2. In this last category, the first study to be analyzed was carried out in 2014, by a group of Serbian scientists that exposed the primary cultures of Leydig cells from Wistar rats to various concentrations of HBCD (0, 1 × 10^−6^, 5 × 10^−6^, and 1 × 10^−5^ mol/L) and analyzed the envisioned effects. They then discovered that HBCD caused a decrease in cellular levels of ATP, inhibited steroidogenesis at various levels, altered cAMP production, cholesterol transport to mitochondria (StAR), conversion of 22-OH cholesterol into pregnenolone (CYP11A1), and the conversion of androstenedione into testosterone (HSD17β) [73].

In the next year, 2015, several scientists performed animal studies in vitro and in vivo involving HBCD. Shi et al. used earthworms to discover the effects of HBCD in vitro and in vivo. The results showed that HBCD had no significant effects on the growth of earthworms, but internally, caused an up-regulation of superoxide dismutase (SOD) levels, suggesting that SOD transcription can be stimulated by oxidative stress. Regarding catalysis (CAT), exposure to HBCD showed no effect on its expression. They also conducted studies on heat shock proteins (Hsps), concluding that the compound significantly increased the expression of the Hsp70 gene [74]. Wu et al. studied Zebrafish embryos and rat cardiomyocyte cell line H9C2 and demonstrated some effects of HBCD at the cellular level. Histopathological analysis of Zebrafish embryos showed that after exposure to the compound, the thickness of the ventricular wall in the hearts had increased. In addition, exposure to HBCD also caused an overload of SR Ca^2+^ (membrane network that acts as a reservoir for Ca^2+^ release) in H9C2 cells. Additionally, Ca^2+^-ATPase activity was also increased, suggesting that calcium activity is affected by HBCD. The expression of miRNA profiles was also altered, with miR-219, miR-145, miR-194b, and miR-221 significantly increased in a dose-dependent manner. MiR-1 and miR-146b were inhibited in zebrafish embryos. In addition, they also found that myocyte enhancer factor 2 (Mef2a) was upregulated in both zebrafish embryo samples and H9C2 cells. With these findings, the authors suggested that the cardiovascular system may be a target of HBCD [75].

In the same year, Genskow et al. performed in vitro and in vivo analyses to assess selective damage to dopaminergic transporters after exposure to HBCD. For the in vitro study, they used dopaminergic primary cultured ventral mesencephalic neurons. The data leads to the conclusion that tyrosine hydroxylase (TH+) dopamine neurons presented sensitivity to HBCD, with significant reductions in a set of neuronal metrics, such as length and arborization of neutrine outgrowths. In addition to the significant loss of TH+ neurons after HBCD treatment, it also caused a reduction in the length and complexity of TH+ neutrine outgrowths involved in communication with adjacent neurons. Experiments conducted in vivo focused on discovering the effects of HBCD on selected presynaptic dopaminergic proteins in the striatum. Exposure of 3-month-old rats to HBCD resulted in a precipitous reduction in the expression of dopamine transporter (DAT) and vesicular monoamine transporter (VMAT2), and these two were integrated into homeostasis mediation and dopamine neurotransmission in the dopaminergic circuit. In contrast, no change in TH expression or dopamine levels in the striatum was observed. These changes occur before explicit damage or even before the loss of the dopaminergic terminal. A reduction in VMAT2 raises important issues over the effects of HBCD on dopamine in the cytosolic compartment of the dopaminergic terminal. The alterations observed by the authors in the striatum seemed to be specific to the dopamine circuit, and there were no alterations in the GABAergic or glutamatergic transporters [76]. Du et al. performed tests on Zebrafish using their liver samples. Exposure to different diastereoisomers resulted in different exposure patterns of hydrocarbon receptors (AHRs) in Zebrafish. These scientists demonstrated that α-HBCD was the most potent in upregulating the expression gene of ahr1a and ahr1b, while γ-HBCD showed a down-regulation on the expression of these genes. A possible explanation for these results would be that diastereoisomers have different binding affinities or distinct interaction mechanisms with AHRs. In addition, HBCD also affected the expression of cyp1a and ethoxyresorufin-O-deethylase (EROD) in a similar way (up-regulation and down-regulation). They also concluded that cyp1a activity is a more bio-sensitive marker to HBCD than EROD activity. They also analyzed the correlation between the expression of AHRs and CYP1s genes. After exposure to α-HBCD, cyp1a was negatively related to ahr1b and positively related to ahr2. The results suggested that the expression of AHRs may be involved in the regulation of CYP1s after exposure to HBCD [77].

In 2016, a group of scientists studied the effects of HBCD using a zebrafish embryo model. Exposure to the compound revealed body malformations and a decreased rate of spontaneous movement in a concentration-dependent manner. They also described an increase in GST activity compared to controls, possibly indicating an increase in oxidative stress. Despite these results, the authors demonstrated that further studies are needed to clarify the role of HBCD [78]. Miller et al. described the changes in EU- and hypo-thyroid female rats after exposure to HBCD. Wistar WU rats were used for this study. On the seventh day of exposure, they observed that there were no alterations in the levels of thyroid hormone (TH) and thyroid-stimulating hormone (TSH). HBCD affected proteins involved in metabolic processes, proteins with specific transport functions, and proteins linked to oxidative stress. Although changes in protein levels were low after short exposure, the same pathways, after one month of exposure, were as affected as short-term exposure. There was also a significant influence of HBCD on gluconeogenesis/glycolysis and amino acid metabolism. In addition, several proteins related to oxidative stress showed alterations. Animals with hypothyroidism demonstrated a very low level of GST compared to EU-rats. The increase in leptin hypo-rats demonstrated a correlation with disturbances in the metabolism of lipids and fatty acids. The authors became interested in performing further studies with exposure to low HBCD concentrations over long periods to confirm whether or not, after a week, the altered proteins are indicative of adverse effects [79].

Additionally, in 2016, Bernhard et al. found it important to describe whether marine fatty acids aggravated the hepatotoxicity of α-HBCD in female rats BALB/c. The differences found in liver histology coincided with changes in liver levels of glutamic acid decarboxylase (GAD). Rats with diets based on marine fatty acids had livers with uniform appearances. Aspartate aminotransferase (AST) and alanine aminotransferase (ALT) were used as markers of liver function and damage. AST levels increased overall in groups. The addition of marine fatty acids resulted in an increase in the expression of peroxisome proliferator-activated receptor α, thus showing that the addition of these fatty acids had an effective functionality in the liver. This addition, together with exposure to HBCD, demonstrated low levels of mRNA in the livers. These results indicate that exposure to α-HBCD has disruptive power in liver metabolism of lipids through β-oxidation leading to increased lipid and steatosis deposition. In addition, marine fatty acids increase the hepatotoxic effect of HBCD. This all shows that diet is a critical variable when we evaluate the effects of HBCD, showing that continued studies are needed to clarify this route of contamination [80]. Miller et al. wanted to specify the gender differences using liver proteome from rats exposed to HBCD. To this end, they used male mice with EU- and hypothyroidism. HBCD did not induce significant differences in T3 and T4 concentrations. They observed that HBCD accumulated less in the adipose tissue of male rats compared to female rats. Mice with hypothyroidism had six altered liver proteins, while Eu-rats had only two altered proteins [81].

In 2018, Shi et al. investigated the expression of antioxidant genes and the metabolic responses of earthworms to HBCD. They found an upregulated SOD, but no changes in GST. After 14 days of exposure several metabolites, such as lactate, glycine, lysine, ATP, and vain, were increased. These results indicate that HBCD may induce anaerobic breathing. The visualized changes in osmotic pressure indicated damage to the membrane structure. Exposure to HBCD also induced oxidative stress [82]. Reffatto et al. used an in vitro and in vivo approach to clarify the effect of HBCD on neurotoxicity. The results showed that the Ca^2+^ and Zn^2+^ pathways were altered, and glutamatergic activity, apoptosis, and oxidative stress. Exposure to low concentrations of HBCD showed that the compound can affect neural signaling in mouse brains by deregulating Ca^2+^ and Zn^2+^ homeostasis [83].

Still, in 2018, Rasinger et al. studied the effects of HBCD on brain proteins and the expression of juvenile female BALB/c mice genes. HBCD demonstrated histopathological effects on the liver, thymus, and uterus. It also showed a decrease in oestradiol 17β (E2) concentrations and an increase in the testosterone/E2 ratio. Proteomic analysis of the brain provided support for the reduction in HBCD-induced E2 levels. The results of this study indicate that exposure to this compound is associated with subtle but relevant toxicological effects [84]. Dong et al. performed studies on *Carassius carassius* to discover antioxidant responses and enzyme biotransformation after exposure to HBCD. Scientists found HBCD very concentrated in fish liver. In addition, it induced the activity of antioxidant enzymes, and increased levels of lipid peroxidation. In conclusion, HBCD induced ecological stress in animals and low levels of biotransformation together with slow bio isomerization rates, suggesting a potential long-term toxic effect [85].

In 2019, Farmahin et al. described the dose–response transcriptional hepatic analysis of male and female Fischer rats after exposure to HBCD. HBCD altered a considerable number of genes involved in xenobiotic metabolism, oxidative stress, immune response, and cell cycle, both in male and female rats. The study supported the idea that HBCD operates through constitutive and pregnane X receptors. Although HBCD demonstrates gender specificity, the pathways it affects are similar in both males and females [86]. Chen et al. describe the multi-generational effects and the stress responses in nematodes after exposure to HBCD. The exposure was conducted in a plate well with 10 mL of treatment solutions (0, 2 × 10^−10^, 2 × 10^−9^, 2 × 10^−8^, and 2 × 10^−7^ mol/L). After exposure, HBCD caused several side effects. They observed that toxicity was inherited. The expression of genes related to oxidative stress and apoptosis was altered. Exposure to the compound could also increase ROS production and cellular apoptosis. The findings of this study were important to evaluate the toxic potential of HBCD [87]. Guo et al. observed the effects on Zebrafish thyroid after exposure to HBCD. The results showed an inhibiting effect of T3 and T4 on the fish liver. Malondialdehyde activity was increased while SOD and CAT activity was decreased. Exposure to HBCD had an inhibiting effect on the thyroid hormone receptor gene. These results showed that the compound can affect the metabolism, transport and generation of thyroid hormones [88].

In 2020, Xie et al. described the effects caused by HBCD on the adipogenesis of C57BL/6 rats. The results demonstrated that after exposure to HBCD, the adipogenic effect increased, showing that HBCD has a dominant role in the first moments of adipogenesis. The mechanism probably involves a specific blockade of the Wnt6 gene during the early stages of adipogenesis. These findings are important to relate HBCD to the development of obesity [89].

In 2020, Bertucci et al. described the cytotoxic effects of HBCD on cultures of fathead minnow liver tissues. The results, involving analysis of genes linked to apoptosis, and genes related to antioxidant responses, revealed that cytotoxicity followed a dynamic pattern, being difficult to describe and characterize the cytotoxicity of this compound [90].

In 2022, Park and Kwak analyzed p53-related apoptotic responses to HBCD in mud crabs. The levels of catalase were increased in the gill and hepatopancreas. The results made it clear that exposure to HBCD significantly increased the transcription levels of p53 in gills and hepatopancreas [91].

Regarding the in vivo studies, in 2015, Heldur Hakk performed studies on rats doing tests on urine, blood and tissues. The authors orally exposed mice to 0.003 g/Kg and observed that this compound accumulated at the tissue level a maximum of 2.5% of the administered dose. The lipophilic tissues contained the highest concentrations, and the α-HBCD was the most favored. These results further support the hypothesis that α-HBCD is the most bio-accumulative and persistent diastereomer when compared to other diastereoisomers. At the excretion level, the author also demonstrated that almost half of the diastereoisomers administered in the animals were excreted in 4 days, with urine being the most important excretive pathway and α-HBCD being the diastereoisomer with less excreting power. Regarding HBCD metabolism, the analyses showed that all diastereoisomers were metabolized, where β-HBCD had the highest metabolized percentage [92]. Bradshaw et al. tried a different approach, testing the effects of HBCD on Mesocosms. The results demonstrated that HBCD can affect coastal ecosystems. The seasonal effects evidenced demonstrated the importance of using experimental conditions relevant to the field under study. The effect of HBCD on the benthic community was probably caused by high and direct exposure to contaminated foods. This effect resulted in changes in the biomass distribution of a key species, such as *M. balthica*, which resulted in changes in nutrient recycling processes. Nevertheless, the authors suggest that it is important to continue to study the problem of chronic exposure of ecosystems to compounds, such as HBCD [93].

In 2016, Wang et al. used the urine of CD-1 rats to metabolically investigate the effects of HBCD exposure. The results showed no symptoms of obvious toxicity or changes in body or body weight. HBCD, however, caused urinary metabolome disturbances after 28 days of continuous exposure and these alterations showed increased citrate and 2-ketoglutarate, along with a decrease in alanine, acetate, formate, trimethylamine (TMA), 3-hydroxybutyrate, and malonic acid. The increase in citrate and 2-ketoglutarate was of great relevance since these metabolites are associated with the tricarboxylic acid cycle (TCA), indicating that the activity of mitochondrial enzymes, related to the TCA cycle, can be activated by HBCD. In addition, they found that HBCD can influence the absorption and use of phenylalanine [94].

In 2017, Ratel et al. studied liver volatolomics from laying hens to reveal their exposure to HBCD. The differences found between the control group and the HBCD-contaminated group show that the compound has the potential to alter metabolism in the liver. They were also able to identify some alkanes and branched alkanes as markers of HBCD contamination. The results showed that the liver volatolome is a good contamination meter through feeding [95]. Szabo et al. described the metabolic profile of the seer in neonatal rats after exposure to HBCD. The results showed stereoisomer-specific metabolic profiles and possible mechanisms of action of HBCD. Phenylalanine was decreased, glutamate was decreased, and arginine. The choline was increased. Ketone bodies were diminished and may be the result of a reduced conversion of acetoacetate to acetate. The authors described that the use of metabolomics is an important and useful approach when we want to study the effect of a compound on the body to help identify biomarkers. [96]

In 2018, Dong et al. analyze the bioconcentration and the effects of HBCD in crucian carp and the effect in the triode hormones concentrations. As a result of exposure (3.12 × 10^−9^, 3.12 × 10^−8^, and 3.12 × 10^−7^ mol/L), the total T3 and total T4 levels were decreased and the activity of acetylcholinesterase (AChE) in the brain was increased. This investigation shows that exposure to HBCD can cause adverse effects on the tested species [97].

Additionally, in 2018, regarding the studies in vitro, Wang et al. studied the effects of HBCD on the liver of *Mossambica tilapia.* The results showed that exposure to HBCD caused a decrease in the activity of EROD, an enzyme used as a biomarker of compounds, such as HBCD [98].

In 2021, Huang et al. found that HBCD could be dehalogenation by CYP168A1 of *Pseudomonas aeruginosa* strain HS9. The results demonstrated that CYP168A1 was up-regulated in response to HBCD. Thus, scientists revealed that CYP168A1 is a new catalytic model of HBCD through debromination and hydrogenation reactions [99]. Additionally, in the same year, Maia et al. described the impact of HBCD on lipid metabolism through an in vitro approach, using the murine 3T3-L1 cell line and the human HepG2 cell line. The results report a decrease in lipid accumulation. In addition, they noticed an increase in monounsaturated fatty acids [100].

In summary, the effects of HBCD in animals are complex and different depending on the animal species. In different animal body parts (liver, thyroid, urine, blood, adipose tissue, and brain), HBCD can induce oxidative stress and cell apoptosis, can affect the cardiovascular system, alter liver metabolism, affect thyroid hormone transport, metabolism, and formation, and increase adipogenic effect, indicating that it may have long-term toxic effects.

**Table 2:** Effects of HBCD in animals.

Study	Study Organism	Exposure	Effects	Reference
In vitro	Juvenile tilapia (Oreochromis mossambicus)	The enzyme supernatant was exposed to HBCD (1.6 × 10^−13^–1.6 × 10^−7^ mol/L)	Decreased the EROD activity.	[98]
	Pseudomonas aeruginosa Strain HS9	1.6 × 10^−6^ mol/L HBCDs mineral salt medium (MSM).	Changed. 277 proteins expression. Upregulated some genes related to heavy metals were. Upregulated cytochrome P450 coding gene cyp168A1.	[99]
	The murine cell line 3T3-L1	Exposure 24 h with HBCD for short-term exposure and exposure with 2.5 × 10^−7^ mol of HBCD for at least 4 weeks for chronic exposure.	Increased lipid accumulation and monounsaturated fatty acids. No alteration of the PPARα expression and the CIDEA expression. Increased PPARγ expression.	[100]
In vitro and in vivo	Wistar Rats	Primary culture of Leydig cells incubated with HBCD in the presence or absence of hCG (1.95 × 10^−10^ mol/L), or with only HBCD (0, 1 × 10^−6^, 5 × 10^−6^ and 1 × 10^−5^ mol/L).	Decreased ATP levels. Inhibition of cAMP accumulation. Decreased transcription of some of the genes involved in steroidogenesis.	[73]
	Earthworms	Cultivation with exposure to HBCD (0, 0.05, 0.1, 0.2 and 0.4 g/Kg dry soil)	Upregulated of SOD and Hsp70. No alteration in CAT levels. No inhibition of growth. Increased oxidative stress in earthworms.	[74]
	Zebrafish embryos and rat cardiomyocyte cell line H9C2	Exposure of the zebrafish to HBCD (0.2 × 10^−9^, 2 × 10^−8^, and 2 × 10^−7^ mol/L). Exposure of the cell line H9C2 to the same concentrations of HBCD.	Increased ventricular wall thickness in the hearts. Increased collagen deposition in the extracellular matrix. Increased atrial and brain natriuretic peptide levels. Cardiac hypertrophy. Disturbed calcium handling.	[75]
	C57BL/6J mice	Orally dose with 0.025 g/Kg of HBCD for 30 days. Ventral mesencephalic neurons cultures with HBCD (0, 1.75 × 10^−6^, 2.5 × 10^−6^, 5 × 10^−6^, 7.5 × 10^−6^ and 1 × 10^−5^ mol/L).	Reduced cell viability. Decreased total number of TH+ neurons and reduction in neurite branching and neurite length of the TH+ neurons. Reduction in the expression of plasmalemmal DAT and VMAT2.	[76]
	Zebrafish	Specific concentrations of specific HBCD diastereoisomers (α- HBCD, β-HBCD and γ-HBCD at 1.6 × 10^−9^, 1.6 × 10^−8^, and 1.6 × 10^−7^ mol/L.	Different expression patterns of the AHRs. α-HBCD and β-HBCD increased the expressions of ahr 1a and ahr1b. γ-HBCD decreased the expressions of ahr1a and ahr1b. Changed EROD activity.	[77]
	Zebrafish	Exposure to HBCD (1.9 × 10^−6^, 3.9 × 10^−6^, 7.8 × 10^−6^, 1.6 × 10^−5^, and 3.12 × 10^−5^ mol/L).	Increased curved body malformations. Decreased rate of spontaneous movement. Increased level of GST activity.	[78]
	Winstar wu Rats	The HBCD was dissolved in corn oil (0.0001 L corn oil added to 0.0009 L custard pudding), and the rats were fed with 0.0005 L of custard pudding. After 4 days of exposure, the rats were orally administered 0, 0.003, and 0.03 g/Kg body weight of HBCD.	Modulation of the proteins involved in metabolic processes and connected with oxidative stress reactions. Decreased hydroxymethylglutaryl-CoA synthase levels. No alteration on gluconeogenesis/glycolysis and the metabolism of several amino acids. No alteration in oxidative stress-related proteins, such as BDH, AL1L1, and ETFB.	[79]
	BALB/c mice	Two different concentrations of α-HBCD (0.0001 and 0.1 g/Kg Body weight per day)	Modulation of the hepatic TAG levels. Increased of the AST levels. Reduced of eiosadienoic acid. Increased of total saturated FAs. Increased the expression of peroxisome proliferator-activated receptor alpha.	[80]
	Rats	Male euthyroid rats (mET) and hypothyroid (mHT) rats were fed with 0, 0.003, and 0.03 g/Kg body weight of HBCD.	Increased corticosterone in mHT. In mET two proteins changes. In mHT 6 proteins changes. In mET male the accumulation of HBCD in adipose tissue was lower than in females. Alterations in lipid metabolism, glycolysis/gluconeogenesis, redox and CYP protein-related responses.	[81]
	Earthworms	Exposure to HBCD (0, 0.05, 0.1, 0.2, 0.4, and 0.6 g/Kg dry soil)	Modulation of the SOD and GST levels. Increased anaerobic respiration. Increased ATP production. Increased of amino acids release. Changes in osmotic pressure.	[82]
	BALB/c mice	Exposure to 0.199 g/Kg body weight per day.	83 genes were altered, 10 upregulated, and 73 downregulated. Decreased in glutamate-dependent Ca^2+^ signaling.	[83]
	BALB/c mice	Exposure to 6 × 10^−9^ g/Kg in the diet.	Increased vacuolation in hepatocytes, lymphocytic infiltration, and hyperaemic vessels. Increased stress in thymus. Increased the reduced density of endometrial glands in uterus. Modulation of four proteins in the brain. Modulation of some proteins, such as MAPK14 and HSP A8.	[84]
	Carassius auratus	Exposure to 3.12 × 10^−9^, 3.12 × 10^−8^, and 3.12 × 10^−7^ mol/L of HBCD.	Decreased TT4 content. Increased AChE activity in brain. Decreased the swimming activity.	[85]
	Fischer Rats	Exposure to 0, 0.25, 1.25, and 5 g/Kg of HBCD.	Modulation of genes expression (involved in metabolism of xenobiotic compounds, steroids and hormones, nuclear receptor activity, cell proliferation, metabolism of glucose and lipids, disruption of the hormonal balance, and oxidative stress).	[86]
	nematode Caenorhabditis elegans	Exposure to 0, 2 × 10^−10^, 2 × 10^−9^, 2 × 10^−8^, and 2 × 10^−7^ mol/L of HBCD.	Transferred effects from the parental generation (F0) to the next (F1). Increased stress-related gene expression in F0. Increased apoptosis and oxidative stress.	[87]
	Liver Zebrafish and zebrafish		After 56 days of exposure: decreased, T3 and T4 in the liver. Decreases the ratio of T3/T4 in the liver first and then increases with the increase in exposure concentration under long-term exposure. Inhibited the malondialdehyde (MDA) activity in low exposure levels and increased in high exposure groups. Increased SOD activity first, then decreases with higher concentrations.	[88]
	3T3-L1 preadipocytes and Hek293 and male C57BL/6 mice	Exposure to HBCD (1 × 10^−7^–1 × 10^−5^ mol/L) of the Hek293 cells. 3T3-L1 preadipocytes were exposed to 1 × 10^−5^ mol/L. Male C57BL/6 mice received oral HBCD (5 × 10^−5^ g/Kg per week)	Adipogenic effect. Increased the expression of adipocyte marker genes Fabp4, PPARγ, Adipoq, and LPL.	[89]
	Liver sections of fathead minnow	Exposure to 4.9 × 10^−10^, 2.5 × 10^−9^, 1.3 × 10^−8^, 6.2 × 10^−8^, 3.12 × 10^−7^, 1.6 × 10^−6^, 7.8 × 10^−6^, 3.9 × 10^−5^, and 1.95 × 10^−4^ mol/L of HBCD.	Down-regulation of caspase2 and apopOn after 6h of exposure. Decreased gene expression of GST, CAT, PI3K and Akt. Decreased enzymes levels involved in xenobiotics metabolism.	[90]
	M. japonicus crabs	Exposure to 1.6 × 10^−9^, 1.6 × 10^−8^, and 1.6 × 10^−7^ mol/L of HBCD.	Increased Catalase expression. Increased Mjp53 expression.	[91]
In vivo	Sprague−Dawley Rats	HBCD was administered orally (0.00725 g/Kg)	The highest concentrations were in lipophilic tissues, adipose tissue, adrenals, skin, and GI tract (>500 ng/g). A total of 50% of the 3 diastereoisomers doses were excreted within 4 days. Urine was the most important excretion pathway. β-HBCD was 80% metabolized, γ-HBCD was 65%, and α-HBCD was 51%. α-HBCD is the most dominant diastereoisomer in biological tissues.	[92]
	Mesocosms	Exposition to 68, 8.5, and 1.1 g/Kg dw of HBCD. To have nine suspensions with a gradient of HBCD, was added the three initial suspensions to obtain various nominal amounts of HBCD per mesocosm (1.3 × 10^−6^, 2.7 × 10^−6^, 5.3 × 10^−6^, 1.06 × 10^−5^, 2.13 × 10^−5^, 4.25 × 10^−5^, 8.5 × 10^−5^, 0.00017 and 0.00034 Kg)	Negative relation between HBCD concentration and the animal biomass Changes in phytoplankton and zooplankton distribution. Dysregulation of the coastal ecosystems.	[93]
	CD-1 mice	Mice were administrated by oral dose with 0.01 and 0.05 g/Kg body weight.	Induced endogenous variations on metabolites, such as increased citrate, 2-ketoglutarate, with decreased alanine, acetate, formate, TMA, 3-hydroxybutyrate, and malonic acid. Modulation of lysine, alanine, and phenylalanine levels.	[94]
	Laying hens	A diet containing 1 × 10^−6^ g/Kg γ-HBCD with laid eggs containing 4 × 10^–7^ g/Kg lw of HBCD.	Increased HBCD levels in tissues. Alcohols, aldehydes and ketones were identified as exposure markers.	[95]
	PND10 mice	Exposure to 0.003, 0.01, and 0.03 g/Kg of α-HBCD, 0.003, and 0.03 g/Kg of γ-HBCD and 0.03 g/Kg of HBCD.	Decreased levels of phenylalanine, glutamate, and arginine. Increased levels of O-phosphocholine and choline. Increased levels of ketone bodies, acetoacetate, and acetone.	[96]
	Carassius Carassius Tent	Exposure to 3.12 × 10^−9^, 3.12 × 10^−8^, and 3.12 × 10^−7^ mol/L of HBCD.	Induced SOD activity. Increased CAT activity. ROS accumulation.	[97]

## 6. Studies in Humans

**Figure 2:**
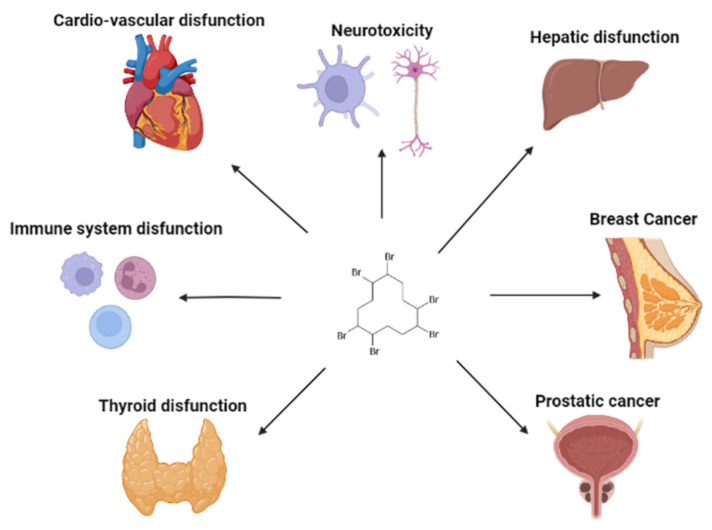
Effects of HBCD in humans, created with BioRender.com (accessed on 26 March 2023).

Concerning the data in humans, summarized in Figure 2, there were only two epidemiologic studies, one in silico and all the others in vitro. For this, this section will also be approached chronologically, where the publication sequence will be the central element of this bibliographic summary.

In 2015, Koike et al. performed a study in vitro to analyze the effect of HBCD on human bronchial epithelial cells. After exposure, the normal human bronchial epithelial cell line (BEAS-2B cell lines) showed that at low HBCD concentrations, there was an increase in cell proliferation compared to controls. On the other hand, cell viability decreased significantly after exposure to this compound. There was also an increase in levels of the intercellular adhesion molecule (ICAM-1), and the cytokines, IL-6, and IL-8. Epidermal growth factor (EGF) production was also significantly increased. After 15 min of exposure, EGF receptor phosphorylation was increased, but the expression of total EGF receptor was not affected. The EGF receptor inhibitor, AG1478, blocked the increase in IL-6 and IL-8 induced by HBCD. Regarding transcriptional nuclear factors, HBCD caused increased activation of nuclear factor-nb (NFκB) p50, p65, and AP-1 c-Jun, but not in signal transducers and activators of transcription (Stat3). These results demonstrated that HBCD has effects on the expression of proinflammatory proteins and on cell viability and proliferation [101].

In the same year, Van den Dungen and some other scientists wanted to study the effects of HBCD related to steroid hormones on H295R cells of adrenocortical carcinoma. After exposure, there was no change in cell viability. HBCD had no significant effects on hormone levels. These findings are in line with what other scientists have previously published, where HBCD has no significant effects on steroidogenesis [102]. An et al. analyze the adaptive responses to HBCD in the immortalized human fetal liver cell line (L02 cells). They then noticed that low HBCD concentrations had no significant effects on cell viability, while higher concentrations decreased cell viability. At low concentrations, HBCD could induce adaptive responses. These responses were regulated by several routes including phosphatidylinositol 3-kinase (PI3K)/protein kinase B (Akt), 5′ AMP-activated protein kinase (AMPK) signaling and via p38 mitogen-activated protein kinases (p38 MAPK). In addition, the chemical structure and metabolic pattern of HBCD could also influence these responses [103].

In 2016, Almughamsi et al. performed studies referring to the changes in the secretion of interferon-gamma (IFN-γ) of human immune cells after exposure to HBCD. After the preparation of peripheral blood mononuclear cell cultures (PBMCs), human monocyte-depleted (MD), and natural killer cells (NK), and their exposure to HBCD, the authors noticed that there was no change in cell viability in any of the cell lines. On the other hand, HBCD caused increased secretion of IFN-γ in NK cells, and in MD-PMBCs and PBMCs. This compound also activated mitogen-activated protein kinases (MAPKs), more precisely the p44/42 pathway, known to regulate the secretion of IFN-γ. In this sense, the authors demonstrated that HBCD has harmful effects on IFN-γ secretion, which can cause inappropriate inflammation [104]. Huang et al. elucidate the effects of HBCD in human hepatoma HepG2 cells and L02 cells. The concentrations of exposure were 0, 10^−7^, 10^−6^, and 10^−5^ M. The studies were conducted using three HBCD diastereoisomers and the authors observed that all the HBCD amylase had toxic effects on both cell lines, inhibiting cell viability, and causing effects on the redox state and DNA damage. The stability of the redox status of HepG2 cells performs an important role in inducing cell toxicity, so the change caused by HBCD would cause serious damage. In conclusion, the authors show that HBCD metabolic rate can influence the cytotoxic effects, and more studies are needed to better describe how the metabolism of this compound can be so harmful [105].

In 2016, Erratico et al. described the metabolism of HBCD diastereoisomers using pooled human liver microsomes and CYP3A4. After incubation of microsomes with HBCD, they observed a varied formation of hydroxylated metabolites. In addition, they noticed that only CYP2B6 and CYP3A4 formed HBCD metabolites in findable quantities, with CYP3A4 being responsible for the formation of more than 80% of metabolites. Thus, the authors were able to conclude that HBCD diastereoisomers form specific and different metabolites, which may be useful for human studies, further suggesting that CYP3A4 performs a very important role in the metabolism of these diastereoisomers [106]. Krivoshiev et al. elucidated the estrogenic effects of HBCD. For this purpose, they used human breast cell line adenocarcinoma, MCF-7. After exposure, HBCD showed significant estrogenic activity, impacting cell viability as well. It also showed that it could act as an estrogen receptor (ER) antagonist, which indicated that HBCD also has anti-estrogenic effects [107].

Additionally, in 2016, Kim et al. describe the influence of HBCD on cell growth regulation, apoptosis and migration in LNCaP prostate cancer cells. The results led to an increase in cell viability after exposure, and an increase in cell proliferation. HBCD has been shown to decrease bax levels, but did not alter bcl-2 levels, indicating that HBCD has apoptotic potential in this cell line. Cell migration capacity was also increased, suggesting that HBCD promotes cell proliferation and migration. Thus, these results together demonstrate the potential of HBCD in the progression of prostate cancer [108]. Anisuzzaman and Whalen described the effects of HBCD on IL-1β secretion using NK cells, MD-PBMCs, and PBMCs. Cell viability after exposure remained unchanged. IL-1β secretion increased in NK cells, and MD-PBMCs and PBMCs. The authors decided to test what would happen if they inhibited Caspase I, which facilitates the production/release of IL-1β. Then, they showed that inhibition of Caspase I occasionally led to inhibition of high IL-1β production caused by HBCD. Nevertheless, in two-thirds of the donors in the study, inhibition of Caspase I did not affect the increased secretion of IL-1β caused by HBCD. These results demonstrated that HBCD has the power to alter IL-1β secretion [109].

Moreover, in the same year, Canbaz et al. described the power of HBCD in activating human monocyte-derived dendritic cells. The results showed that exposure to HBCD led to the activation and production of cytokine, increasing the expression of human leukocyte antigen—DR isotype (HLA-DR), CD86, and IL-8. HBCD led to the maturation and activation of dendritic cells. Despite these findings, the authors also reported the need to conduct more studies to discover the contribution of HBCD in the development of immune allergic responses [110].

In 2017, Li et al. described also in vitro studies of the genotoxicity of HBCD in cultured human breast cells through DNA damage. Using HBL-100 cells, the authors found that, with the increase in HBCD concentration, the rate of cell proliferation decreased, and cell viability. ROS production increased at high HBCD concentrations and lactate dehydrogenase (LDH) increased by a concentration-dependent trend, indicating that HBCD produced cytotoxic damage to cells damaging their membranes. The breast cancer gene 1 (BRCA1) expression was also promoted with HBCD increase, which showed a prognostic of breast cancer in the cell line. They were then able to conclude that HBCD induced oxidative stress by two-ways: the first by breaking DNA by ROS, and the second through cells with unrepaired DNA, which can lead to cell death. Despite these findings, the mechanisms of cell death and DNA repair cession need to further studies [111]. In the same year, a study analyzed the changes in the secretion of tumor necrosis factor-alpha (TNFα) of human immune cells after exposure to HBCD. NK cells, MD-PBMCs, and PBMCs were used. The data demonstrated that TNFα secretion increased in NK cells, MD-PBMCs, and PBMCs, after exposure to HBCD. To try and understand if the effect of HBCD could be countered, several inhibitors, such as c-Jun N-terminal kinase (JNK), MAPK extracellular signaling-regulated kinase kinase (MEK), NFkB, metalloprotease TNFα converting-enzyme (TACE), matrix metalloproteinases (MMP), and p38 of TNFα production pathways were also studied. One of the pathways used, the p38 MAPK pathway, was of great importance so that HBCD could increase the secretion of TNFα, thus contributing to chronic inflammation [112]. Jin et al. decided to use L02 cells to see if HBCD promoted autophagy via PI3K/Akt/mammalian target of the rapamycin (mTOR). The results show that HBCD increased cell apoptosis of L02 cells. The LC3-I and LC3-II proteins involved in autophagy were significantly elevated after exposure to HBCD. Incubation of cells with HBCD showed high levels of proteins related to the pathway under study, leaving evidence that HBCD can activate this pathway and promote autophagy in L02 cells [113].

In 2019, Xie et al. conducted an in vitro study on human preadipocytes from visceral adipose (HPA-V) to describe the promotion of adipogenesis after exposure to HBCD. The results showed that exposure to HBCD did not alter cell viability, but promoted the formation of lipid droplets, still increasing the lipid content. HBCD also elevated adipogenic genes, but without statistical significance. All these data together demonstrated that HBCD could promote adipogenesis in human preadipocytes [89]. Shi et al. describe the neurotoxicity of HBCD in human SH-SY5Y neuroblastoma cells. In this study, all HBCD diastereoisomers caused inhibition of cell viability, increased cell permeability, and disassembly of actin fibers, thus causing cytotoxicity in the cells under study. Apoptotic bodies were found in the samples after exposure. The levels of ROS and SOD were high. One of the main toxicity mechanisms observed in this study appeared to be caspase-dependent apoptosis. Treatment with HBCD also led to oxidative stress, which is likely to be responsible for the apoptosis of SH-SY5Y cells [114].

Additionally, in the same year, the impact of HBCD on lipid metabolism was studied. For this purpose, they used the murine cell line 3T3-L1 and the human cell line HepG2. The 3T3-L1 cell lines showed no changes in proliferation, but after exposure to HBCD showed a decrease in lipid accumulation. In HepG2, lipid accumulation increased. These results suggest that HBCD may have a major impact on lipid accumulation in hepatocytes [100].

Recently, in 2022, Sousa et al. also performed an in vitro study and described the effect of HBCD on MCF-7 cells through the impact on the vitamin D pathway. The cell migration was decreased after exposure. After 4 weeks of exposure to HBCD, cells demonstrated a different response to calcitriol, associated with possible DNA damage. These results show that the response of the cell cycle to vitamin D changed, and may indicate DNA damage performed by HBCD, and further studies are needed to clarify the mechanisms of action [115].

In 2019, Orgono et al. described, through an epidemiological study, exposure to HBCD and the risk of type 2 diabetes (T2D) in the French E3N cohort. The data showed that dietary exposure to HBCD was linearly associated with an increased risk of T2D, and the main food groups that contributed to greater exposure to HBCD were non-processed white meat and processed meat. This is the first epidemiological study to find a linear relationship between this compound and the risk of developing T2D, demonstrating the importance of better investigating the effects and risks of this compound for human health, and further studies are needed to confirm this discovery [116].

In the same year, Huang et al. also performed an epidemiological study in the population of Beijing, China, to detect the HBCD values in breast milk. The goal was also to discover the levels of contamination, temporal trends, and risk assessment. HBCD has been found in almost all the samples, with levels between the limit of detection (set at zero) and 3.63 × 10^−5^ g/Kg, which were much higher than other Chinese cities, suggesting that Beijing has a very rich HBCD pollution. The authors also showed that HBCD values may continue to rise in human milk and in biotic and abiotic matrices [117].

In 2021, Zainab et al. wanted to describe the role of HBCD in the progression of breast cancer and the identification of alpha estrogen receptor inhibitors through in silico studies. The results showed that HBCD may be involved in the prevailing of breast cancer and may be one of the major contributors to its appearance given its interaction with the Erα protein. Thus, the authors conclude that for these results to be validated, in vitro and in vivo studies will be necessary [118].

In summary, the data present in this review demonstrate that this compound has harmful effects on human health (Table 3). This flame retardant induces a decrease in cell viability to increase cytokine production, increases the production of pro-inflammatory proteins, induce adaptive responses, has estrogenic activity, promotes cell migration and proliferation, contributes to chronic inflammation, and even causes DNA damage. On the other hand, the epidemiological study demonstrated that dietary exposure to HBCD was linearly associated with a T2D risk increase. Moreover, the link between exposure to this compound and the increase in breast cancer also seems clear and more large-scale populational epidemiological studies are needed.

## 7. Conclusions

In conclusion, HBCD and its substitutes can affect the entire environment in different ways. Despite the various restrictions already imposed on these compounds around the world, their prevalence in the food chain eventually affects plants, animals, and human systems. These compounds can be inherited and are accumulated in blood, urine, and breast milk. Several studies have found that these EDCs can affect several systems, such as the neuronal, cardiovascular, or endocrine system, and/or liver volatomics, by causing DNA damage, altered proteins, cytokine production, and inducing chronic inflammation. Currently, there is still only one epidemiological study with a positive correlation between high levels of HBCD and the risk of Type 2 diabetes. Despite this, to ascertain the pathways that HBCD substitutes use to cause toxicity, there is still a need for further epidemiological studies, in vivo, in vitro, and in silico. Thus, it is urgent to carry out more studies to evaluate the effect of these compounds on human health and the ecosystem, to prevent human pathologies and possible treatments for the most adverse effects of HBCD.

## Figures and Tables

**Table 1:** Effects of HBCD on plants.

Study	Study Organism	Exposure	Effects	Reference
In vitro	Maize roots and shoots	HBCD—0, 3.12 × 10^−9^, 7.8 × 10^−9^, 1.6 × 10^−8^, 3.12 × 10^−8^ and 7.8 × 10^−8^ mol/L	Roots present the highest HBCD levels. Inhibition of germination rate, root biomass, root elongation, shoot biomass, and shoot elongation.	[69]
	Maize roots	α-HBCD—3.9 × 10^−11^ mol/L, β-HBCD—2.7 × 10^−11^ mol/L and γ-HBCD—3.12 × 10^−12^ mol/L	Presence of the three enantiomers: α-, β-, and γ-HBCD in the maize roots.	[70]
	Pak Choi leaves	Seeds were put into glass pots with Hewitt’s nutrient solution and HBCD	Alteration on several metabolic pathways.	[71]
In silico and in vitro	Maize	Microsome prepared with 10 × 10^−3^ mol/L of technical HBCD	Decreased and alteration in the protein content of maize CYPs.	[72]

**Table 3:** Effects of HBCD in humans.

Study	Study Organism	Exposure	Effects	Reference
In vitro	normal human bronchial epithelial cell line, BEAS-2B	Exposure to 1.6 × 10^−8^–1.6 × 10^−5^ mol/L of HBCD	Increased cell proliferation at low concentrations and decreased at higher concentrations. Increased ICAM-1 expression, IL-6 and IL-8 production. Increased EGF production. EGFR-specific tyrosine kinase inhibitor (AG1478), blocked the HBCD-induced IL-6 production.	[101]
	H295R human adrenocortical carcinoma cells	Exposure to 1 × 10^−6^ mol/L of HBCD to cytotoxicity tests.	No alteration in the hormone levels.	[102]
	The human hepatic cell line L02	Exposure to 10^−13^, 10^−11^, and 5 × 10^−5^ mol/L of HBCD	Suppressed cell survival. PI3K/Akt pathway, AMPK signaling, and p38 MAPK pathway regulation.	[103]
	PBMCs and monocyte-depleted PBMCS/NK cells	Exposure to HBCD (5 × 10^−8^ to 5 × 10^−6^ mol/L) for 24 h, 48 h, and 6 days.	Increased IFN-γ secretion from NK, PBMC, and MD-PBMC cells. Decreased the secretion of some enzyme inhibitors.	[104]
	Human hepatoma HepG2 cells and L02 cells	Exposure to 0, 10^−7^, 10^−6^, and 10^−5^ mol/L of HBCD.	No inhibitory effects. Intracellular redox state and DNA damage alterations. Increased ROS levels.	[105]
	pooled human liver microsomes	Exposure to 1 × 10^−8^ to 1 × 10^−4^ mol/L of HBCD.	Increased hydroxylated metabolites formation. rCYP2B6 and rCYP3A4 formed HBCD metabolites.	[106]
	The human breast adenocarcinoma cell line, MCF-7	Exposure to 1 × 10^−4^ mol/L of HBCD.	Increased estrogenic activity. Modulation of the cell viability.	[107]
	LNCaP prostate cancer cells	Exposure to 10^−8^–10^−5^ mol/L of HBCD.	Increased LNCaP cell proliferation and migration. Increased cyclin D1 expression.	[108]
	NK cells/PMBC and monocyte-depleted (MD) PBMC	Exposure to HBCD (5 × 10^−8^ to 5 × 10^−6^ mol/L) for 24 h, 48 h, and 6 days.	Increased IL-1β secretion from NK, MD-PBMC, and PBMC cells. Some enzyme inhibitors could decrease the secretion of IL-1β caused by HBCD.	[109]
	Peripheral blood mononuclear cells (PBMC)	Exposure to 0.1 × 10^−7^, 1 × 10^−6^, 1 × 10^−5^, and 2 × 10^−5^ mol/L of HBCD	Enhanced CD86 expression. Increased IL-8 production.	[110]
	human breast cells HBL-100	Exposure to 0, 7.8 × 10^−6^, 1.6 × 10^−5^, and 7.8 × 10^−5^ mol/L of HBCD.	Low concentrations increased proliferation rate, contrarily to higher concentrations. Increased ROS production and the DNA tail. BRCA1 was promoted with HBCD increase, which exhibited a prognostic of breast cancer.	[111]
	PBMCs, and monocyte-depleted PBMCS/NK cells	Exposure to HBCD (5 × 10^−8^ to 5 × 10^−6^ mol/L) for 24 h, 48 h, and 6 days.	Decreased TNFα secretion from NK cells. Increased TNFα secretion by MD-PBMC cells and PBMC cells.	[112]
	L02 cell line	Exposure to HBCD (5 × 10^−8^ to 5 × 10^−6^ mol/L) for 24 h, 48 h, and 6 days.	Increased cellular apoptosis. Increased LC3-I (initial step of autophagy) and LC3-II proteins. Increased PI3K/Akt/mTOR pathway. Increased autophagy.	[113]
	3T3-L1 preadipocytes and Human/Preadipocytes from visceral adipose (HPA-V)	Exposure to 2 × 10^−5^ and 4 × 10^−5^ mol/L of HBCD.	Increased lipid droplets formation. Induced adipogenesis during the first 2 days.	[89]
	SH-SY5Y human neuroblastoma cell line	Exposure to 1 × 10^−10^, 1 × 10^−9^, and 1 × 10^−8^ mol/L of HBCD.	Decreased cell viability. Increased cellular apoptosis and necrosis. Increased ROS level.	[114]
	The murine cell line 3T3-L1/The human cell line HepG2	Incubation of 24 h with HBCD for short-term exposure and long-term exposure incubation, at least for 4 weeks, was used 2.5 × 10^−7^ mol/L of HBCD.	Decreased cell proliferation. Reduced MMP-9 expression and cell migration. Long-term exposure induced an uncommon response to calcitriol	[100]
	MCF-7 cells/HepG2 cells	Incubated for 24 h with HBCD (1 × 10^−8^ to 5 × 10^−7^ mol/L). Long-term exposure, for at least 4 weeks, used 2.5 × 10^−7^ mol/L HBCD.	Decreased cell proliferation in a dose-dependent manner. Reduced the expression of MMP-9 and cell migration. Long-term exposure induced an uncommon response to calcitriol.	[115]
Epidemiological	French E3N cohort	71,415 women, of which 3667 were (type 2 diabetes) T2D cases	Dietary exposure to HBCD was linearly associated with a T2D risk increase. Non-processed white meat and processed meat were the main groups that contributed to HBCD dietary exposure.	[116]
	Breast milk	111 individual samples were obtained.	HBCD was detected in almost all samples and the concentrations were between the limit of detection that was set as zero and 36.3 n/g lw	[117]
In silico	Breast cancer		Involved in breast cancer prevailing. Showed strong interactions with the Erα protein.	[118]

## Data Availability

Not applicable.
